# Endoscopic Resection and Topical 5-Fluorouracil as an Alternative Treatment to Craniofacial Resection for the Management of Primary Intestinal-Type Sinonasal Adenocarcinoma

**DOI:** 10.1155/2010/750253

**Published:** 2011-02-16

**Authors:** Simon Mackie, Tass Malik, Hisham Khalil

**Affiliations:** ^1^Peninsula Medical School, Derriford Hospital, Plymouth, PL6 8DH, UK; ^2^Department of Otolaryngology, Derriford Hospital, Plymouth, PL6 8DH, UK

## Abstract

*Introduction*. Intestinal-type adenocarcinoma of the sinonasal tract is very rare and is responsible for less than 4% of tumours of the sinuses. Craniofacial resection has been the mainstay of treatment for many years; however, techniques for endoscopic resection are constantly being developed. *Discussion*. The use of transnasal endoscopic resection (TER) and topical chemotherapy applications as an alternative to cranio-facial resection (CFR) is discussed. TER offers advantages over CFR in terms of fewer intra-operative complications and an improved cosmetic outcome. Survival and metastatic rates are similar between both procedures. Patients with locally invasive tumours are better managed with CFR. Topical applications of 5-Fluorouracil has been shown to be effective in increasing survival in patients with sino-nasal malignancy. *Conclusion*. Trans-nasal endoscopic resection and topical 5-Fluorouracil could potentially offer an acceptable alternative treatment to the standard of cranio-facial resection. This should be investigated in trials with a longer followup period than this paper in order to directly compare the two treatment modalities.

## 1. Introduction

Adenocarcinoma of the paranasal sinuses is rare and generally follows an aggressive clinical course [1]. Craniofacial resection has been the mainstay of treatment for many years now and represents the gold standard of surgical resection. However, endoscopic techniques for resection are constantly being developed and may represent a viable alternative with fewer complications. As well as this, chemotherapy and radiotherapy are important treatment options and can be used to augment the effects of surgery [2].

In this paper, a patient with sinonasal adenocarcinoma treated by endoscopic resection is presented.

## 2. Case Report

**Figure 1:**
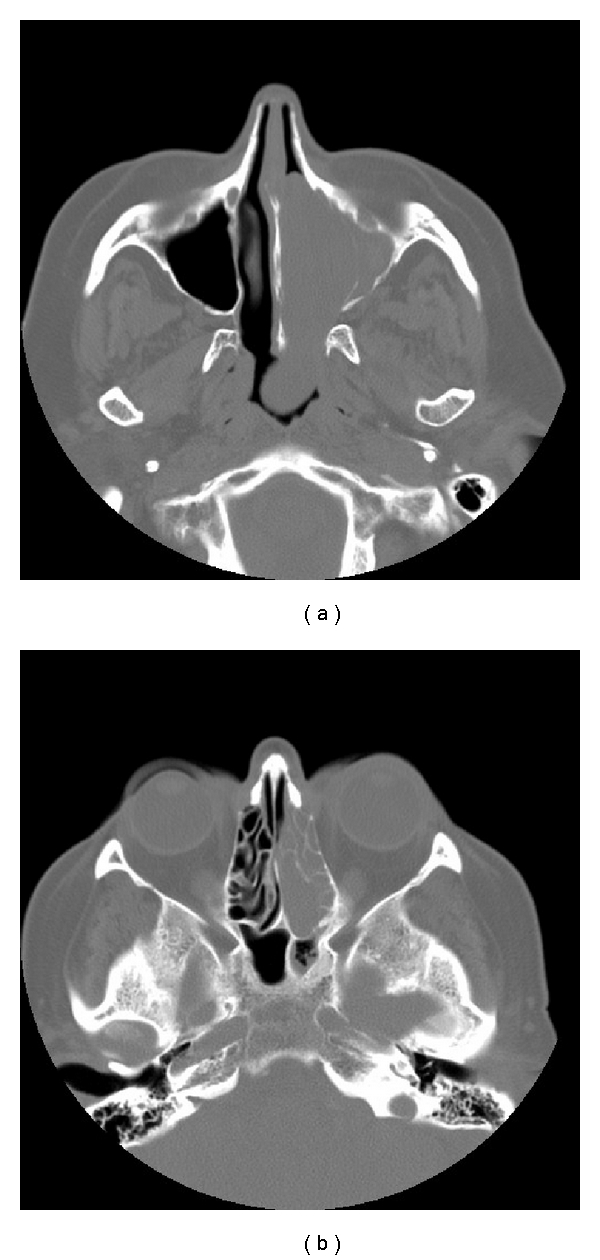
CT scans taken in 2006 after the patient's initial referral to ENT. (a) CT scan showing a mass in the left nasal cavity, and (b) CT scan showing the mass in the left ethmoid sinus, The mass was initially thought to be an inverted papilloma, but following histology it was shown to be an intestinal-type adenocarcinoma.

A 66-year-old woman presented for biopsy of the left ethmoid sinus and nasal cavity in order to assess possible recurrence of ethmoidal adenocarcinoma. The patient was initially referred 3 years ago to the Ear Nose and Throat (ENT) Department by her G.P. after she experienced continual unilateral left facial pain. On biopsy of the left ethmoid sinus, there was shown to be an intestinal type adenocarcinoma. MRI imaging of the face/neck and CT of the thorax and abdomen displayed that the malignancy was confined to the left paranasal sinuses (Figure 1). 

**Figure 2:**
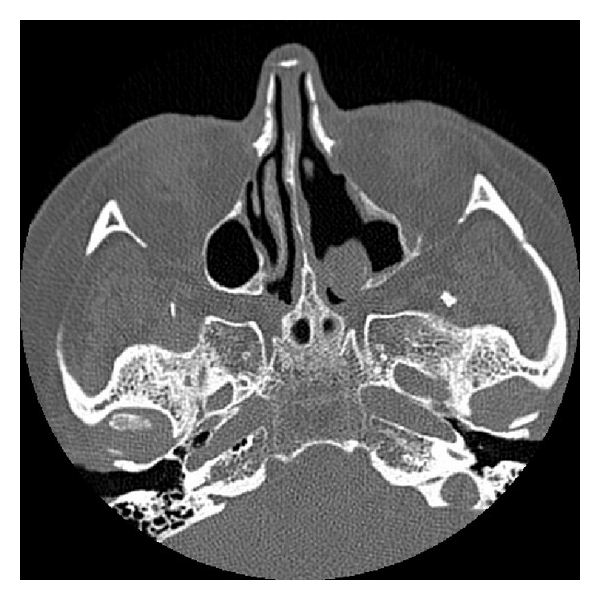
CT scan taken in 2007 showing evidence of surgery in the patient's left nasal cavity. The lesion has been removed, and the maxillary ostium is widened.

**Figure 3:**
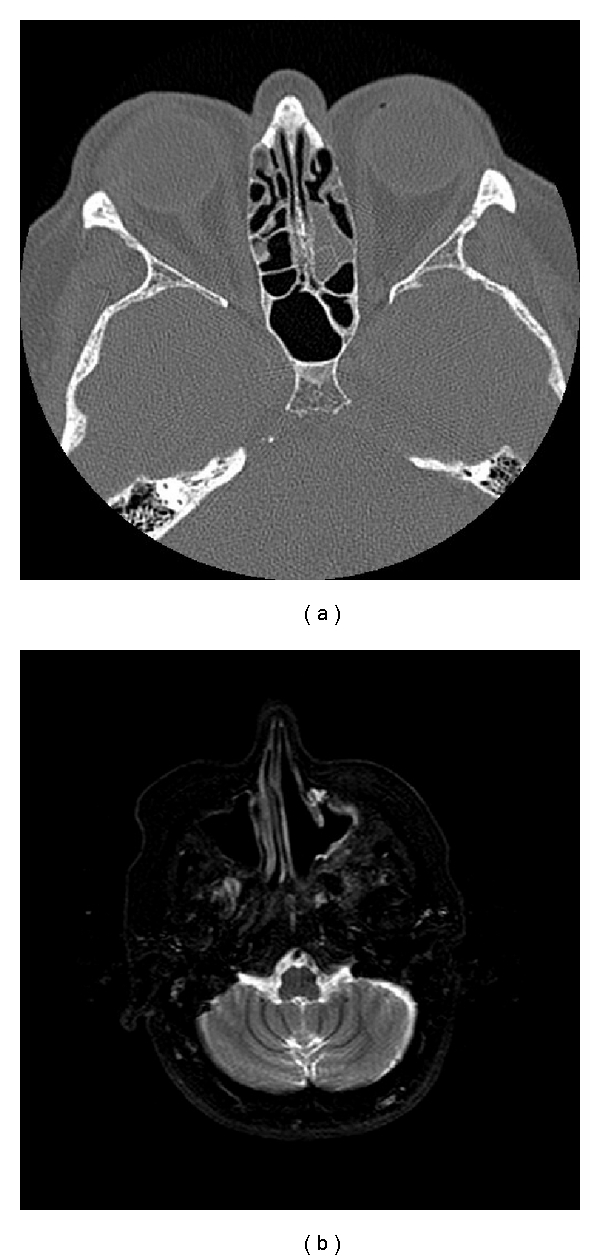
(a) CT scan taken in 2007 showing soft tissue with bony wall destruction in the patient's left posterior ethmoid sinus, which was a recurrence of the malignancy and treated with topical chemotherapy. (b) MRI scan taken in 2008 showing an area of high signal in the patient's left maxillary antrum, indicating the need for a biopsy to be performed.

Despite being offered a craniofacial resection (CFR), for cosmetic reasons, the patient elected for endoscopic removal of the tumour, which was carried out in August 2006 (Figure 2). However, the patient continued to experience left-sided dull facial pain, and on biopsy a recurrence of the tumour was found (Figure 3). This was treated with a further endoscopic resection.

Following this, regular biopsies were taken, and residual disease following the revision endoscopic resection was treated with 5-Fluorouracil topical chemotherapy. Topical chemotherapy was again used in September 2008 to treat a recurrence of the tumour, located in the posterior ethmoidal sinus. 

The patient has continued to experience dull left-sided facial pain and due to this presented for biopsy. The biopsies show no evidence of neoplasia, and the patient will return for regular followup.

## 3. Discussion

A review of the literature was carried out using databases such as Medline, Embase, and the Cochrane Library. The references of review articles were also used. 

### 3.1. Craniofacial Resection

Craniofacial resection (CFR) was first described in 1963 and has since been considered the standard treatment for malignancies involving the anterior skull base [2, 3]. Whilst CFR has been shown to have low recurrence rates, it is also associated with a long recovery and complication rates as high as 40% in some studies [2].

### 3.2. Is Endoscopic Resection a Viable Alternative?

Endoscopic resection as a technique to remove malignant anterior skull base lesions has always been controversial, with many arguing that it is not safe. However, in the light of recent advances in both technology and equipment, techniques are now available which allow this resection to be carried out safely. Transnasal endoscopic resection (TER) is a procedure that many now believe to be a reasonable alternative to CFR [2].

Several studies have aimed to compare the effects of both CFR and TER. Eloy et al. performed a retrospective analysis in Florida which found that there were no significant differences in complication rate, postoperative survival, or metastasis between the two procedures. Moreover, it found that hospital stays were significantly shorter in the TER group of patients. Finally, improved cosmetic outcome was found with endoscopic resection. This is an important aspect for many patients, and TER can completely eliminate the need for external incisions and therefore scars [2]. This view that TER is safe and advantageous is found in many studies [2, 4–6].

TER also holds advantages intraoperatively. The excellent visualisation offered by the endoscope could result in a safer and more precise procedure. Podboj and Smid. found that operating time was on average an hour shorter, and blood loss during surgery was less than half of that with traditional external approaches. As well as this, postoperative radiotherapy, which may be delayed by wound healing with CFR, can be administered immediately following TER [5].

Endoscopic resection for sinonasal tumours is reported to be a demanding procedure. Several papers support the view that it is a safe technique in the hands of a skilled and experienced surgeon [2, 5]. Aside from this patient, selection is an important issue. Patients with tumours invading the orbit, skin, or lateral recess of the frontal sinus are better managed with conventional CFR [4].

One potential problem with TER is the effect on surgical excision margins. This approach usually results in a piecemeal resection approach, and this means that normal histopathological methods of measuring excision margins may not be possible. As a result of this, it is suggested that samples for frozen section are to be taken during the surgery to be analysed postoperatively [5]. As well as this, inadequate resection margins may make recurrence more likely, and it is suggested that this is more common with an endoscopic approach [7].

Recurrence of the malignancy postoperatively for both endoscopic and traditional methods has been measured in several studies. Recurrence is the primary cause of cancer-related death in patients with ethmoidal malignancies and therefore is an important aspect of any treatment [8]. One recent study found no significant difference between the methods in terms of metastatic and 5-year survival rates. There was a slight increase in survival with endoscopy; however, one explanation for this was the higher rate of advanced cancers treated with CFR as opposed to TER [2]. Aside from this, the increased visualisation made possible by utilizing endoscopy, as opposed to the limited visualisation with CFR, could also be responsible for this slight difference and represents a possible significant advantage of TER. 

Complications are associated with both CFR and TER techniques. Complications involving the central nervous system such as Cerebral-Spinal Fluid leaks and pneumocephalus can be encountered in both procedures. However, the lack of brain retraction with an endoscopic approach reduces the possibility of brain contusion and edema, which can occur with CFR [4]. Nevertheless, studies which have directly compared the procedures found no difference in complication rates, with the one possible difference being increased blood loss with CFR [2].

Aside from pure endoscopic resection, endoscopic-assisted CFR has gained acceptance as a standard procedure for management of sinonasal malignancies. This approach allows for a combined transfacial and transnasal technique, resulting in a single external incision. This also avoids the limited working angle which is possible with an endoscopic transnasal approach alone. A combined technique may broaden the cases for which endoscopy is suitable [5, 7].  Table 1 displays an overview of the key advantages and disadvantages of a Transnasal Endoscopic Approach.

### 3.3. The Role of 5-Fluorouracil

5-Fluorouracil is an antimetabolite and can be used to treat a variety of cancers, including head and neck cancers. It can be used both intravenously and topically. Toxicity is unusual, with myelosuppression and cerebellar syndromes being rare complications. Its use in sinonasal malignancy is usually topical following surgery, and this along with other forms of topical chemotherapy are recognised forms of treatment [9, 10].

A large trial in Rotterdam aimed to assess whether a traditional CFR approach was superior to surgical debulking followed by the application of topical chemotherapy. Survival rates were measured over a 23 year period using the Kaplan-Meier method and a significant increase in survival was found in patients treated with debulking and topical chemotherapy. It suggests that this method becomes the mainstay of treatment for sinonasal malignancy [9].

A further study in the UK directly compared survival rates between 5-Fluorouracil chemotherapy and traditional methods of radiotherapy and CFR. It found that the survival rates of 50% with these traditional methods improved to 86% with initial surgical intervention followed by topical chemotherapy. This is a significant difference and supports the view that topical 5-Fluorouracil treatments following surgery become standard for the management of sinonasal malignancy [11].

## 4. Conclusions

Transnasal endoscopic resection could represent an acceptable treatment for patients with sinonasal malignancy that decline the gold standard treatment of craniofacial resection. It is gaining a reputation as a safe and effective treatment. Topical chemotherapy has been shown to increase survival when combined with debulking surgery and is also an effective treatment option.

It must also be remembered that TER is not acceptable for patients with locally invading tumours, and patients must be selected carefully. Further trials should be undertaken to directly compare these treatment modalities. Although this case report supports positive outcomes up to four years post-operation emphasis should be placed on long-term followup of patients in order to allow further comparison.

## 5. Summary

What is already known on this topic is the following:

- craniofacial resection is the standard of treatment for sinonasal Malignancies;
- endoscopic resection has always been controversial.

What this paper adds on the topic is the following:

- transnasal endoscopic resection could represent an acceptable treatment for patients who decline a craniofacial resection;
- the addition of topical chemotherapy to endoscopic resection can improve survival;
- further trials including long-term followup directly comparing the two treatment modalities are required.

## Figures and Tables

**Table 1:** Key advantages and disadvantages of transnasal endoscopic resection.

Advantages	
(i) Better cosmetic outcome	
(ii) Shorter operating time	
(iii) Shorter hospital stay	
(iv) No delay in postoperative radiotherapy	

Disadvantages	
(i) Must be performed by a highly skilled and experienced surgeon	
(ii) Not suitable for patients with local invasion or advanced malignancies	
(iii) Difficulty determining surgical excision margins	
